# Potential vectors of *Trypanosoma evansi* in the Canary Islands (Spain): diversity, abundance, and climatic and site-related factors

**DOI:** 10.1186/s13071-026-07443-9

**Published:** 2026-05-13

**Authors:** Adrián Melián-Henríquez, Juan Alberto Corbera, Margarita González-Martín, Sergio Martín-Martel, Manuel Morales-Doreste, Joel Israel Moo-Millan, Marc Desquesnes, Geoffrey Gimonneau, María Teresa Tejedor-Junco

**Affiliations:** 1 Research Institute of Biomedical and Health Sciences, University of Las Palmas de Gran Canaria, Canary Islands, Las Palmas de Gran Canaria, Spain; 2 Hospital Clínico Veterinario, Universidad de Las Palmas de Gran Canaria. Canary Islands, Las Palmas de Gran Canaria, Spain; 3 Laboratorio de Parasitología, Centro de Investigaciones Regionales “Dr. Hideyo Noguchi”, Universidad Autónoma de Yucatán, Mérida, México; 4 UMR INTERTRYP, CIRAD, 34398 Montpellier, France; 5 INTERTRYP, Univ Montpellier, CIRAD, IRD, Montpellier, France; 6 CIRAD, ENVT, 31300 Toulouse, France; 7 Laboratoire National de l’Elevage et de Recherches Veterinaires, Institut Sénégalais de Recherches Agricoles, Hann, BP 2057, Dakar, Senegal

**Keywords:** *Stomoxys*, Hippoboscidae, Tabanidae, Surra, Vector ecology, Mechanical transmission

## Abstract

**Background:**

*Trypanosoma evansi* is a protozoan parasite responsible for surra, a disease of significant veterinary and economic impact. Mechanical transmission by haematophagous Diptera, particularly *Stomoxys* spp. and Tabanidae, plays a central role in its spread. The Canary Islands represent a relevant setting for this research, as several historical outbreaks of surra have been reported in local dromedary populations. This study aimed to assess the diversity and abundance of potential *T. evansi* vectors in the province of Las Palmas (Canary Islands, Spain), with particular emphasis on the species *Stomoxys calcitrans*, and to evaluate the influence of climatic and site-related factors on their distribution.

**Methods:**

Over a 2-year period (2023–2024), dipteran specimens were sampled using Nzi and Vavoua traps at ten locations across Gran Canaria, Lanzarote, and Fuerteventura. Morphological identification and environmental data collection were performed at each site. Generalised linear mixed models (GLMMs) were used to assess the influence of climatic and site-related parameters on species abundance.

**Results:**

A total of 23,695 haematophagous Diptera were collected, with *S. calcitrans* being the predominant species (99.6%). *Pseudolynchia canariensis* (0.4%) and *Tabanus cordiger* (a single specimen) were also detected. GLMMs revealed variations in *S. calcitrans* abundance, specifically in relation to mean temperature, mean wind speed, distance between traps and dung heaps, and trap type.

**Conclusions:**

These findings provide a baseline for the ecological surveillance of mechanical vectors of *T. evansi* in island ecosystems, with particular reference to the Canary Islands. Further longitudinal studies are needed to assess seasonal dynamics and potential vector-host interactions under local conditions. However, given its high abundance, *S. calcitrans* appears to be the predominant species of potential epidemiological relevance and should be prioritised in surveillance and control strategies under the conditions studied.

**Graphical Abstract:**

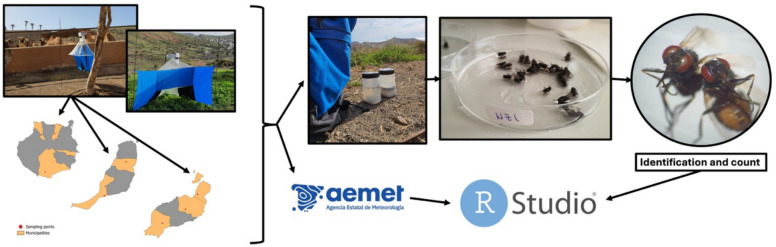

## Background

Trypanosomatids (Order: Kinetoplastida) are a diverse group of flagellated protozoa that infect a wide range of hosts, including insects, plants, and vertebrates [1, 2]. Among them, the genus *Trypanosoma* is of particular importance because several species are pathogenic and cause major diseases in both humans and animals [3–6]. While most species are transmitted by haematophagous insect vectors, alternative transmission routes, such as oral, venereal, and iatrogenic pathways, have also been documented [3].

*Trypanosome* species are traditionally classified into two major groups, *Salivaria* and *Stercoraria*, based on the anatomical site of development within the insect vector [7]. Salivarian trypanosomes undergo cyclical development in the anterior part of the vector’s digestive tract and are transmitted biologically through biting mouthparts or saliva. In contrast, Stercorarian species complete their development in the hindgut and are transmitted via faecal contamination [8, 9].

A key representative of the *Stercoraria* group is *Trypanosoma cruzi*, the causative agent of Chagas disease in humans, which is transmitted by triatomine bugs (Hemiptera: Reduviidae) [10, 11]. On the other hand, the *Salivaria* group includes African trypanosomes such as *Trypanosoma brucei gambiense* and *T. b. rhodesiense*, responsible for human African trypanosomosis (also called sleeping sickness), as well as *T. vivax*, *T. congolense*, and *T. b. brucei*, which cause animal trypanosomosis (also known as nagana) [12, 13].

While tsetse flies (*Glossina* spp., Diptera: Glossinidae) are the primary biological vectors of African trypanosomes, *Trypanosoma evansi* (Chauvrat, 1896), the etiological agent of surra, represents a notable exception. This parasite does not undergo cyclical development in vectors and is instead transmitted mechanically by various haematophagous dipterans, relying on the direct transfer of infective trypomastigotes through the mouthparts of biting flies during interrupted blood meals [3].

Mechanical vectors involved in the transmission of *T. evansi* include biting flies of the genus *Stomoxys*, the family Tabanidae, and the family Hippoboscidae, among others [3, 14]. The efficiency of this mode of transmission is influenced by the parasite load in the host’s blood and by the vector’s behaviour, particularly its tendency to switch hosts within short time intervals (typically < 30 min), during which viable parasites may persist on the insect’s mouthparts [3]. The diverse behavioural and ecological traits of these insects may influence their capacity for transmission. In general, members of the family Tabanidae are commonly associated with humid and semi-humid habitats (river margins, pastures, forest edges, etc.), as oviposition typically occurs near water bodies [15]. Adult activity is predominantly diurnal, with peaks in abundance during summer months or during rainy seasons in tropical areas [15, 16]. Species of the genus *Stomoxys*, particularly *Stomoxys calcitrans* (Linnaeus, 1758), are considered cosmopolitan and are typically associated with livestock environments, including pastures and farm facilities [15]. Their larvae develop in decomposing organic matter, and adult populations exhibit marked diurnal activity, with peaks during warmer months, while in humid tropical regions, their activity may be prolonged [17, 18]. In contrast, in hippoboscids, as obligate ectoparasites of birds and mammals, their distribution and seasonal dynamics are largely determined by their hosts and host behaviour [15, 19].

From a genetic perspective, the loss of maxicircle kinetoplast DNA (kDNA) in *T. evansi* has been proposed as a key evolutionary adaptation to mechanical transmission. This genetic modification renders the parasite unable to complete its developmental cycle within tsetse flies, effectively eliminating the need for a biological vector [20].

This protozoan was first reported in Spain in 1997 following the detection of trypomastigotes in a dromedary (*Camelus dromedarius*) imported from Mauritania to Gran Canaria [21]. Subsequent investigations confirmed the presence of the parasite in other islands of the Canary archipelago, including Lanzarote and Fuerteventura [22]. Its introduction into mainland Europe was later attributed to the movement of infected animals from the Canary Islands, with outbreaks documented in both continental Spain and France [23, 24].

In the Canary Islands, additional studies were carried out to identify potential reservoirs among domestic livestock. Although isolated cases of seropositivity were detected, parasitological and molecular diagnostic tools failed to confirm the presence of *T. evansi* in equines or ruminants [25, 26]. To explore the persistence of infection within affected dromedary herds, two complementary studies were undertaken based on distinct hypotheses. The first investigated wild rodents as possible reservoirs but detected only *Trypanosoma lewisi*, thus ruling out their involvement in the epidemiological cycle of surra [27]. The second study focused on entomological factors and reported the exclusive presence of the haematophagous fly *Stomoxys calcitrans* in one of the herds with the highest prevalence of disease, suggesting its potential role in the transmission and maintenance of the parasite [28]. The implementation of a structured *T. evansi* surveillance and control programme by the Department of Agriculture, Livestock, Fisheries, and Food Sovereignty of the Canary Islands Government in 2017 resulted in no newly detected positive cases [29, 30].

Within the framework of the European COMBAT project (Controlling and progressively Minimizing the Burden of Animal Trypanosomosis) [31], the present study aimed to assess the diversity and abundance of potential *T. evansi* vectors (with particular emphasis on the species *S. calcitrans*) and to evaluate the influence of climatic and site-related factors on their distribution on selected islands of the Canary archipelago that have been historically affected by surra. Given the lack of studies on the ecology of these insect vectors in the Canary Islands, the data generated in this study provide a first comprehensive insight into the presence of haematophagous Diptera on livestock farms across several islands and contribute essential epidemiological information to support ongoing entomological surveillance, refine early detection protocols, and inform the design of long-term vector control and disease prevention strategies in insular settings.

## Methods

### Study area

**Fig. 1:**
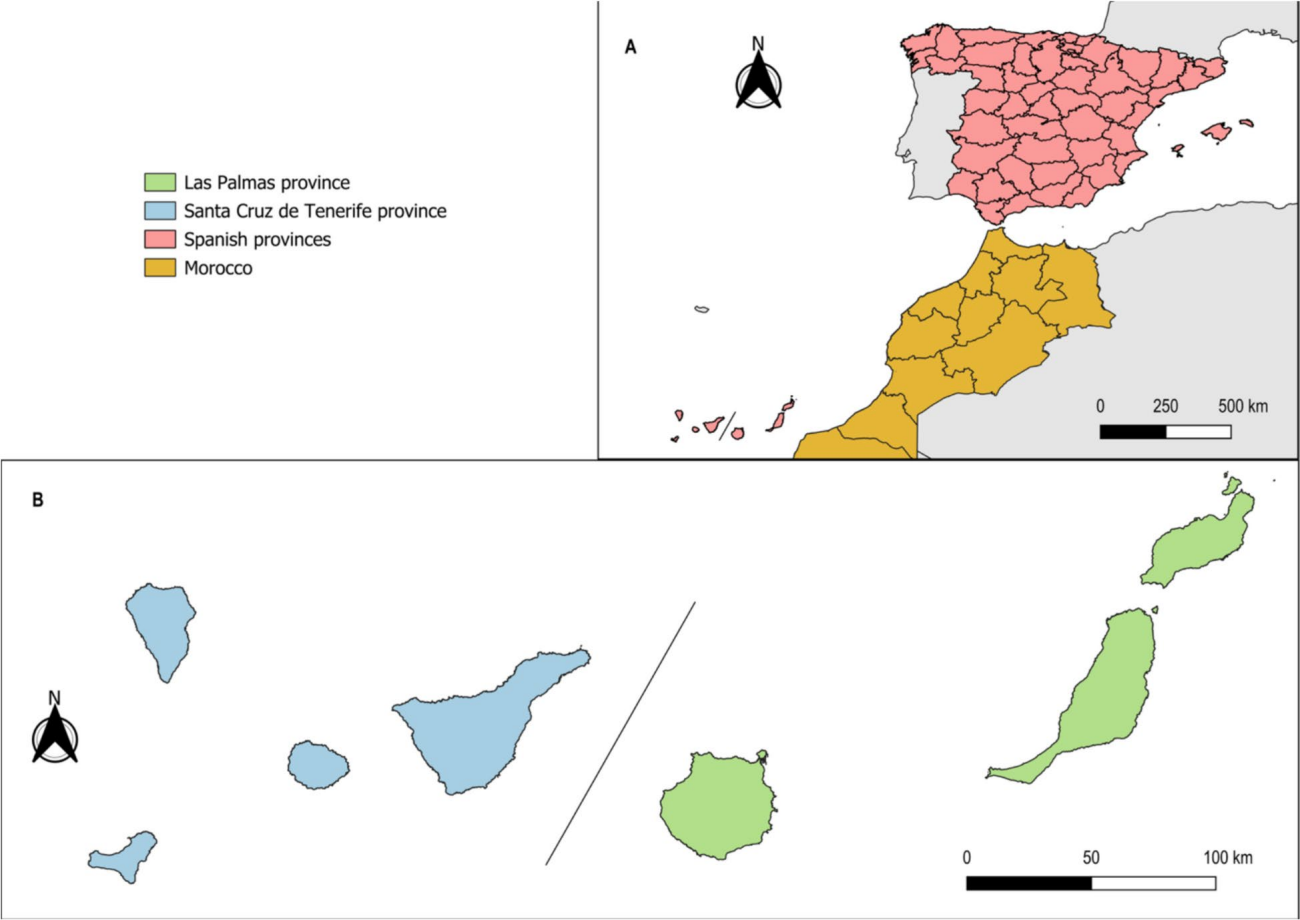
Geographical location of the Canary Islands

This study was conducted in the Canary Islands, Spain, an archipelago located in the Atlantic Ocean, northwest of the African continent. Specifically, the islands of Gran Canaria, Fuerteventura, and Lanzarote (in the province of Las Palmas) were selected for sampling (see Fig. 1).

The selection of sampling locations, primarily livestock farms, was based on a combination of epidemiological and ecological criteria. The three islands included in the study host most of the dromedary population in the archipelago. Given that historical outbreaks of surra in the Canary Islands have been predominantly associated with this species, proximity to farms housing dromedaries was established as a key selection criterion. Sites where the presence of other potential hosts of *T. evansi* (including small ruminants, cattle, and equids) has been confirmed were also incorporated. Overall, locations selected in the southern regions of the islands either hosted dromedary farms or were near them, whereas in northern areas, the aforementioned livestock species predominated.

Beyond host presence, an effort was made to select locations representative of the different climatic zones on each island, with a primary distinction between the northern and southern regions. The Canary Islands are generally characterized by a stable, dry climate influenced by trade winds [32]. However, the term “microclimate” is often used to describe the region’s climatology, as no island exhibits a fully homogeneous climate pattern. According to Köppen’s climate classification system (1936), several subclimates have been identified across the archipelago (Spanish State Meteorological Agency [AEMET], 2012). Overall, on the islands of Lanzarote and Fuerteventura, approximately 90% of their surface area is characterized by Type B climates (warm desert) according to the Köppen classification. In contrast, Gran Canaria, largely due to its complex orography, exhibits a more heterogeneous climatic pattern. Type B climates predominate in coastal areas, with warm desert conditions mainly in the south and warm steppe conditions in the north. In contrast, Type C climates occur in higher altitude areas of the north-central part of the island.

**Fig. 2:**
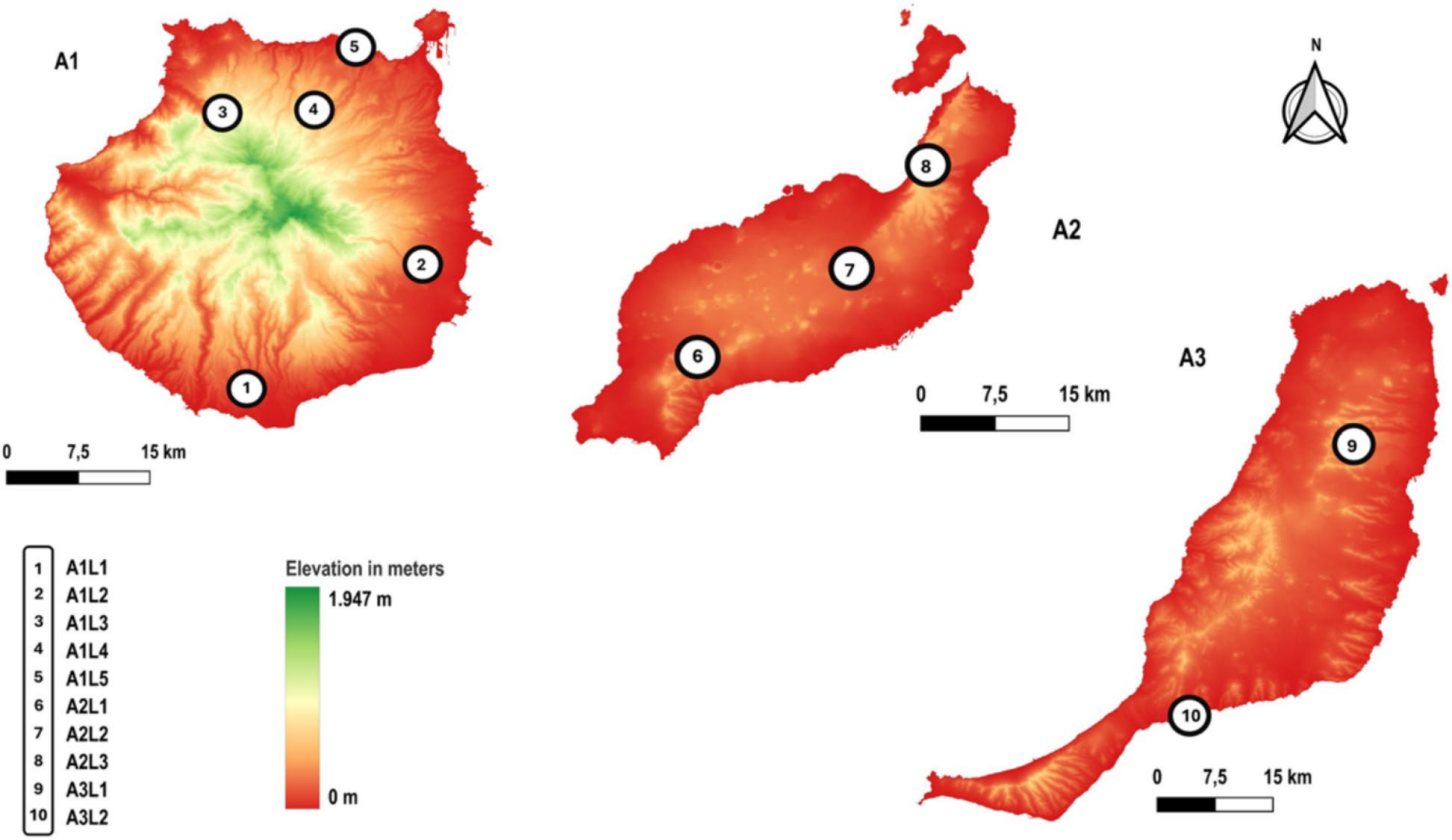
Sampling sites in the province of Las Palmas. A1: Gran Canaria Island; A2: Lanzarote Island; A3: Fuerteventura Island

Five locations were surveyed in Gran Canaria, three in Lanzarote, and two in Fuerteventura. Each sampling point was coded using a combination of the island (A) and the specific location (L) (see Fig. 2).

Regarding other relevant characteristics of these sampling sites, low-altitude areas (particularly in southern regions and coastal zones) were characterized by halophytic and xerophytic vegetation. In contrast, higher altitude areas, especially in the north-central regions of Gran Canaria, were characterized by grasslands and shrublands, as well as other angiosperm vegetation and cultivated fields.

The proximity of the sampling sites to populated areas varied among islands. In Gran Canaria, the mean distance was approximately 1 km (± 200 m), except for site A1L1, which was only 300 m from a populated area. In Fuerteventura, the average distance was 850 (± 100) m, whereas in Lanzarote, it was 1.9 km (± 100 m), except for site A2L2, which was 3 km from the nearest populated area.

Before site selection, farm owners and caretakers were consulted regarding the use of insecticides, and none reported using them. Additionally, they were asked about the location of dung accumulations on each farm, as this substrate is crucial for the larval development of certain vector species. This information was considered when positioning the traps.

### Sampling methodology

Once the farms included in the study had been selected according to the previously described epidemiological and ecological criteria, the most suitable trap configuration and expected performance at each location were assessed. Trap placement was guided by three main criteria: visibility of the animals from the installation point, a minimum distance between traps, and the absence of direct visual contact among them.

The first criterion aimed to enhance trap detectability by haematophagous dipterans, as placement in overly sheltered areas (such as behind structures or within dense vegetation) could reduce trapping efficiency. The criteria of minimum spacing and lack of inter-trap visibility were adopted to minimize potential interference in captures. Although the study was not designed to formally compare the different trap types used, trap distribution across distinct sectors within each farm was intended to optimize the capture of haematophagous dipterans.

A minimum separation distance of 50 m was established [33], considering the spatial constraints of the selected farms, while ensuring, as far as possible, that traps were not visible to one another from their respective positions. Although these conditions could not be strictly met in all cases because of space limitations, they were generally upheld in most locations.

Initial trap performance was evaluated through a preliminary sampling conducted in October 2022. This trial lasted 7 days and confirmed the capture of haematophagous dipterans in all installed traps. Although differences in the number of specimens captured were observed (mainly among trap types), these variations were not considered sufficiently limiting to warrant modifications to the selected sampling sites. Consequently, the initial configuration of locations was maintained, and the study’s sampling programme was subsequently initiated.

Entomological surveys were conducted between January 2023 and October 2024. At each site, traps were deployed for 8 to 10 consecutive days, with sampling conducted quarterly (January, April, July, and October) to capture potential seasonal variation. Two Vavoua traps and one Nzi trap (ZeroFly^®^), all unbaited, were installed at each sampling site.

At two locations, deviations from this standardised protocol were necessary. At location A1L5, situated in an urbanized area with limited available space, only two Vavoua traps were deployed. Conversely, at location A3L2, which hosted a large dromedary population and had ample space, an additional Vavoua trap was added to increase the sampling effort.

### Trap setup

Devices were installed using iron rods of varying lengths (1.8 m, 1.3 m, and 0.5 m), semi-rigid PVC tubes for Nzi traps, and galvanized wire for structural support. The specimen collection system consisted of plastic bottles and cans joined using hot-melt silicone. After deployment, approximately 300 mL of 70% ethanol was added to each trap’s collection container to preserve specimens. In locations or periods with high ambient temperatures, this volume was increased to 400 mL to compensate for evaporation.

### Climatic variables

Climatic variables (including mean temperature, mean relative humidity, accumulated precipitation, and mean wind speed) were recorded monthly throughout each sampling period to evaluate their potential influence on the abundance of haematophagous dipterans. Meteorological data were retrieved from the Spanish State Meteorological Agency (AEMET) via its official online platform (https://x-y.es/aemet/ca-canarias), using the nearest available stations to each sampling site.

Specifically, meteorological stations on the island of Gran Canaria were an average distance of 3.5 km (± 1 km) from the selected locations, except for site A1L4, where the station was only 500 m away. In contrast, stations on the islands of Fuerteventura and Lanzarote were generally located approximately 10 (± 2) km from the locations, except for site A2L3, where the distance was 2 km.

### Insect identification

Insects were identified using standard dichotomous keys [15, 19, 34–36] under a stereomicroscope (VWR VisiScope^®^, model SZB260). Haematophagous dipterans were identified to the species level, while non-haematophagous insects were identified only to higher taxonomic ranks. The analysis focused primarily on haematophagous species, with systematic recording of sex (male/female).

Post-prandial specimens were frequently observed across most sampling periods. These individuals were preserved in 70% ethanol at − 20 ºC for potential future morphological or molecular analyses.

### Data analysis and software

To assess the data distribution (specifically, abundance per trap and sampling period for the species *S. calcitrans*), normality was evaluated using the Shapiro-Wilk and Bartlett tests. Because the data did not meet the assumption of normality, nonparametric statistical methods were used. Generalised linear mixed-effects models (GLMMs) with a quasi-Poisson distribution (link = “log”) were selected, based on the best fit obtained from Pearson’s residuals. Prior to model fitting, Spearman’s rank correlation was used to examine potential multicollinearity among climatic variables. The models were developed at the island level, except for Gran Canaria, where northern and southern zones were analysed separately because of differences in topography and environmental conditions. In the models, the identification number of each trap was used as a random effect, while mean temperature, mean wind speed, trap type, and distance to dung accumulations were treated as fixed effects.

All analyses were carried out using RStudio (version 2024.12.1 Build 563; © 2009–2025 Posit Software, PBC). The packages used included *readxl* for data import [37], *dplyr* for data manipulation [38], *nortest *[39] and car [40] for testing model assumptions, *lme4* [41] for implementing statistical models, and *MASS* [42] and *ggplot2* [43] for graphical representations. A *P*-value < 0.05 was considered statistically significant. Model explanatory power was evaluated using marginal and conditional *R*^*2*^s [44], which quantify the variance explained by fixed effects and by the full model (fixed + random effects), respectively. The package used for this purpose was *MuMIn *[45].

## Results

### Overall results

A total of 23,695 haematophagous dipterans were captured over the study period, with 46% collected in 2023 and 54% in 2024. The specimens belonged predominantly to one species, *Stomoxys calcitrans* (Linnaeus, 1758; Diptera: Muscidae), commonly known as the stable fly, and secondarily to a few specimens of *Pseudolynchia canariensis* (Macquart, 1839; Diptera: Hippoboscidae), an avian ectoparasite. Additionally, a single female of *Tabanus cordiger* (Meigen & Wiedemann, 1820; Diptera: Tabanidae) was collected during preliminary sampling in 2022 at site A3L2. No further individuals from this species (or any other Tabanidae) were recorded in subsequent samplings.

Notable differences were observed in the total number of *S. calcitrans* and *P. canariensis* captured. A total of 23,596 *S. calcitrans* were recorded, with a sex ratio of 63% males to 37% females. In contrast, only 99 specimens of *P. canariensis* were collected, with 61% males and 39% females. Due to the considerable disparity in abundance between the two species (and given that *P. canariensis* is an obligate ectoparasite of birds), subsequent statistical analyses and island-specific results focused exclusively on *S. calcitrans*. Results are presented separately for each island. Although each location displayed specific characteristics and no fully consistent patterns were evident, several common trends emerged across sites. In general, capture peaks occurred most frequently in April, followed by January, July, and October; however, the patterns showed variability between years within the same location and among different locations.

In addition to the two focal species, a total of 35,332 non-haematophagous specimens belonging to the family Muscidae were captured throughout the sampling period. Occasionally, individuals belonging to other insect orders were also collected. A detailed list of non-haematophagous taxa is provided in Table 1, although these represented a minor proportion of the total sample.

**Table 1:** Orders and families of identified non-haematophagous insects occasionally encountered in samplings

Order	Families
Coleoptera	Buprestidae, Cerambycidae, Coccinellidae, Curculionidae, Staphylinidae
Dermaptera	NI
Diptera	Asilidae, Bombyliidae, Calliphoridae, Drosophilidae, Lauxaniidae, Muscidae, Psychodidae, Sarcophagidae, Sepsidae, Syrphidae
Ephemeroptera	NI
Hemiptera	Lygaeidae, Miridae, Pentatomidae
Hymenoptera	Andrenidae, Apidae, Tiphiidae, Vespidae
Lepidoptera	NI
Neuroptera	Chrysopidae
Orthoptera	Acrididae

NI: family level not identified. Abundance data not included

### Gran Canaria

**Fig. 3:**
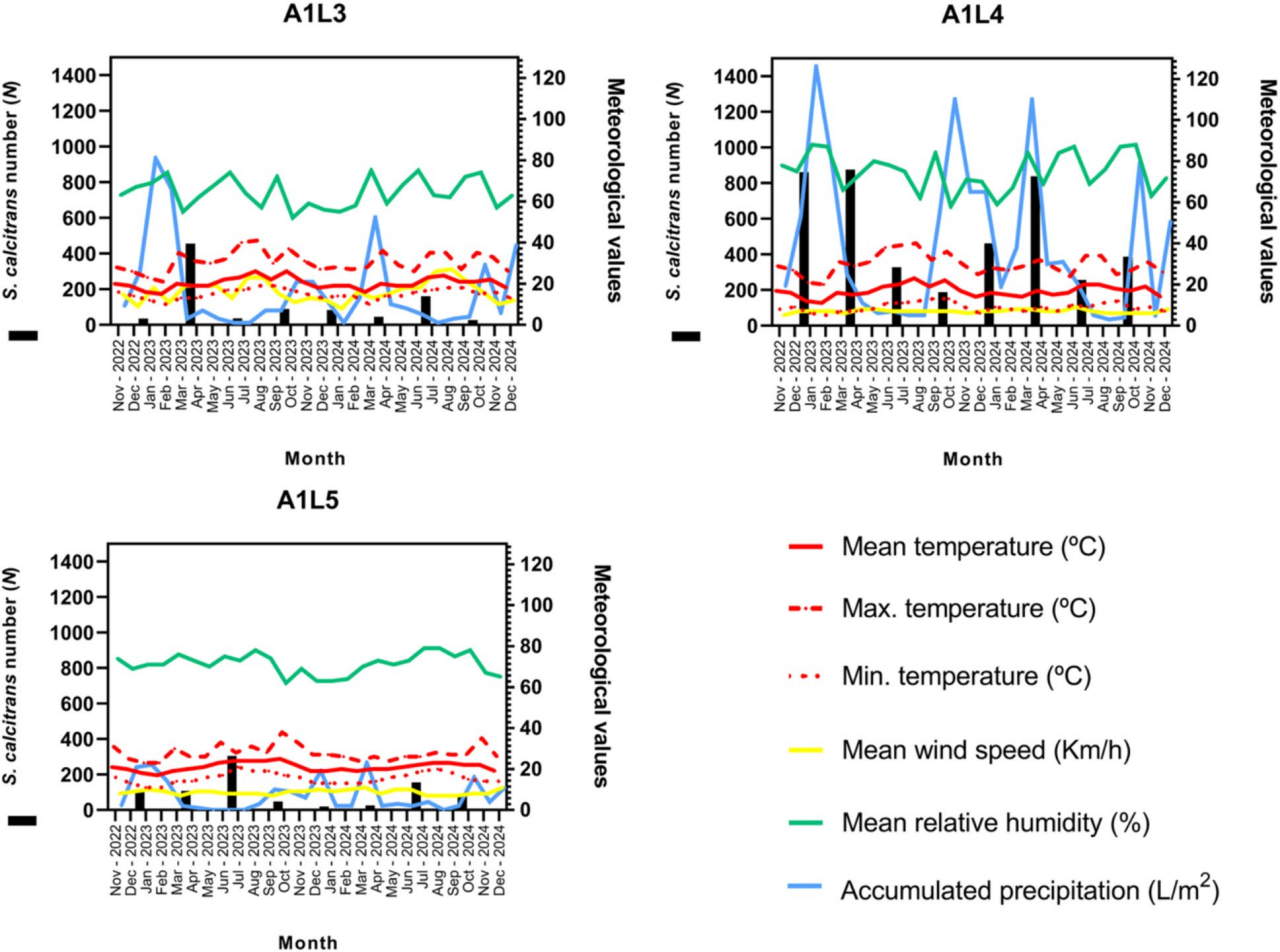
Number of *Stomoxys calcitrans* specimens and meteorological data in northern locations on Gran Canaria (2023–2024)

**Fig. 4:**
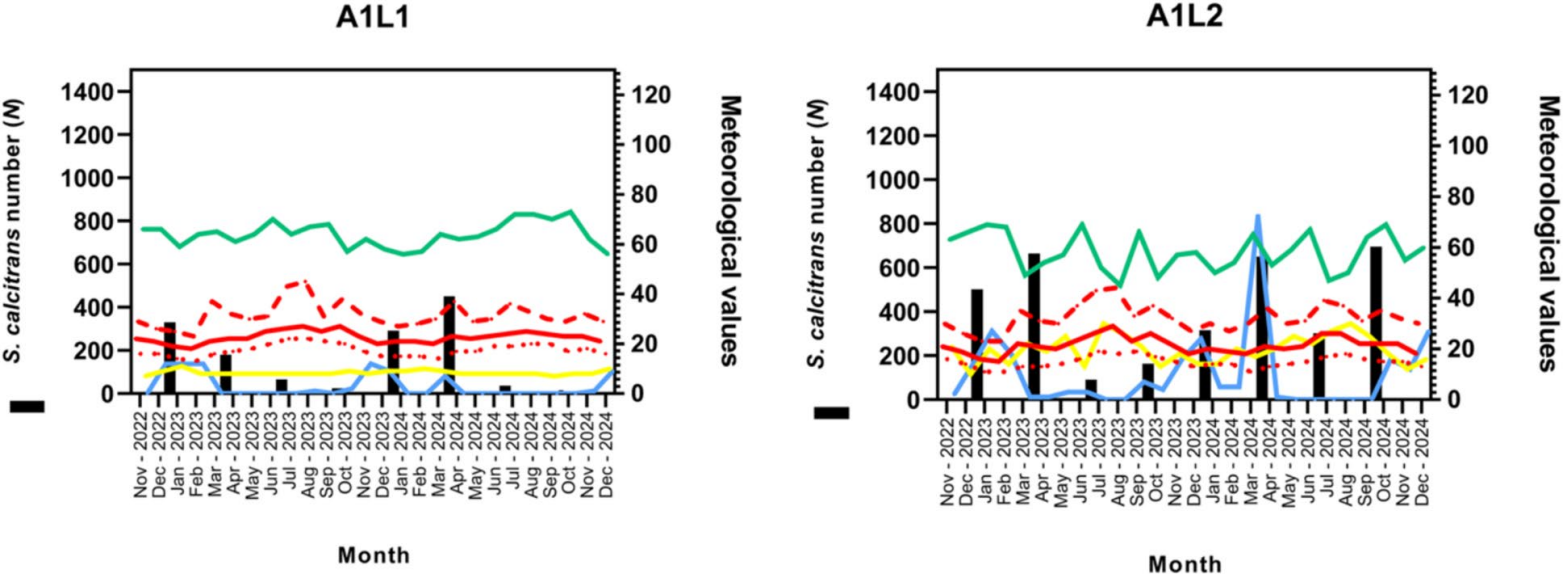
Number of *Stomoxys calcitrans* specimens and meteorological data in southern locations on Gran Canaria (2023–2024)

On Gran Canaria, 10,774 *S. calcitrans* individuals were captured, with a sex ratio of 60% males to 40% females. Additionally, 43 *P. canariensis* specimens were recorded (65% males, 35% females), along with 7576 other Muscidae. The site with the most *S. calcitrans* captures was A1L4, accounting for 39% of the total, followed by A1L2 (31%), A1L1 (13%), A1L3 (9%), and A1L5 (8%). Temporal variation in captures and meteorological data across the sampling period is shown in Fig. 3 (north locations: A1L3, A1L4, and A1L5) and Fig. 4 (south locations: A1L1 and A1L2).

At site A1L1, *S. calcitrans* captures in 2023 peaked in January and declined steadily through October. In 2024, however, the peak shifted to April, followed by a progressive decrease until October.

At A1L2, the highest count in 2023 occurred in April, followed by a marked drop in July and a rebound in October. In 2024, April again showed high numbers, but the peak occurred in October after a notable mid-year decline.

At A1L3, the seasonal pattern differed between years: in 2023, the peak was in April, with a decline in July and a slight recovery in October; in 2024, the highest count was observed in July, with a sharp decrease by October.

At A1L4, a consistent trend was noted across both years: high counts in January, peaks in April, and decreases in July. However, whereas October 2023 showed a continued decline, October 2024 exhibited a resurgence in captures.

Finally, A1L5 displayed a uniform pattern over both years, with steadily increasing captures up to a peak in July, followed by a decline in October.

### Lanzarote

**Fig. 5:**
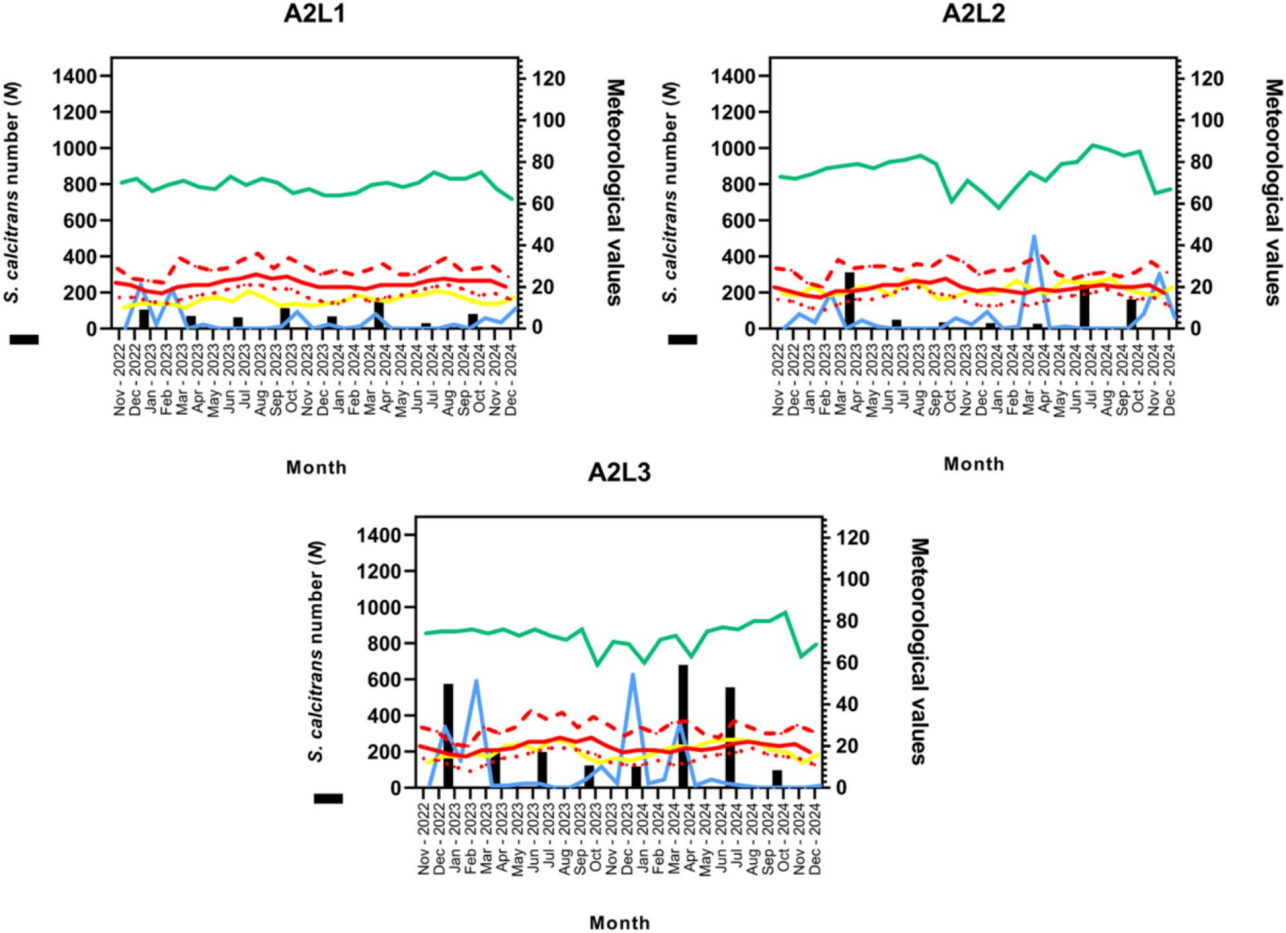
Number of *Stomoxys calcitrans* specimens and meteorological data in locations on Lanzarote (2023–2024)

In Lanzarote, a total of 4123 *S. calcitrans* were captured (59% males, 41% females), along with 5 specimens of *P. canariensis* (60% females, 40% males) and 23,158 other Muscidae. The site A2L3 accounted for the most *S. calcitrans* captures (62%), followed by A2L2 (21%) and A2L1 (17%). Temporal variation and meteorological data across sampling periods are shown in Fig. 5.

At A2L1, 2023 exhibited a gradual decline in captures from January to July, followed by a marked increase in October. In contrast, in 2024, the peak occurred in April, followed by a sharp drop in July and an increase in October.

At A2L2, captures in 2023 rose from just a few individuals in January to a peak in April, then declined in July and October. In 2024, counts remained low in January and April, peaked in July, and declined again in October.

At A2L3, captures peaked in January 2023, then decreased in April, remained stable in July, and declined further in October. The 2024 pattern differed, with low counts in January, a peak in April, a slight decline in July, and a sharp drop by October.

### Fuerteventura

**Fig. 6:**
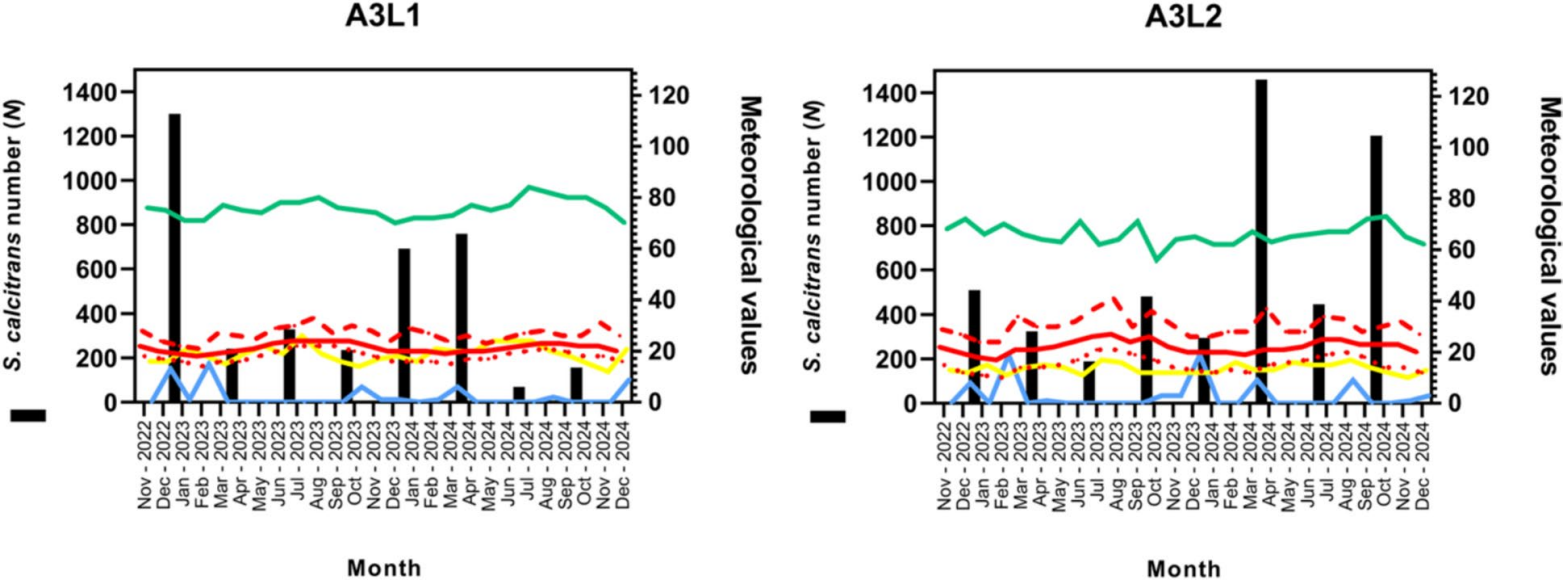
Number of *Stomoxys calcitrans* specimens and meteorological data in locations on Fuerteventura (2023–2024)

On Fuerteventura, 8699 *S. calcitrans* specimens were collected (69% males, 31% females), along with 51 *P. canariensis* (59% males, 41% females) and 4598 other Muscidae. The site A3L2 recorded the most *S. calcitrans* captures (57%), followed by A3L1 (43%), both contributing significantly to the island’s totals. Temporal variation and meteorological data are shown in Fig. 6.

At A3L1, 2023 showed a peak in January, followed by a sharp drop in April, a minor increase in July, and a further decrease in October. In 2024, the pattern shifted: high numbers in January, peaking in April, and declining in July, with a slight rebound in October.

At A3L2, the 2023 peak occurred in January, followed by a steady decline until July and a recovery in October. In 2024, the lowest counts were recorded in January, followed by a peak in April, a decline in July, and an increase again in October.

### Environmental and sampling factors

The potential influence of environmental and methodological factors on the abundance of *S. calcitrans* was evaluated. Variables included mean temperature, mean relative humidity, accumulated precipitation, mean wind speed, trap type, and distance to nearby dung accumulations. Due to significant correlations between temperature and both relative humidity (Spearman’s *rs* = −0.38, *P* < 0.001) and precipitation (*rs* = −0.41, *P* < 0.001), only temperature and wind speed were retained as climatic predictors in the GLMMs.

In Gran Canaria, the GLMM for the northern sites (Table 2; Model 1) showed a significant negative effect of temperature on *S. calcitrans* abundance, indicating that higher temperatures were associated with a marked reduction in captures (*P* < 0.001). Wind speed also showed a significant negative effect, although weaker than that of temperature (*P* = 0.03). In contrast, the trap type had a strong positive effect, with the Vavoua trap capturing considerably more individuals than the reference trap (*P* = 0.002). In addition, distance from dung accumulations showed a significant negative relationship with abundance, indicating that captures decreased as the distance from the dung heap increased (*P* = 0.04).

**Table 2:** Model 1: GLMM results for locations in northern Gran Canaria, assessing the effect of mean temperature, mean wind speed, trap type (Nzi as reference vs. Vavoua), and distance between traps and dung accumulations on the number of *Stomoxys calcitrans* specimens collected per trap

	Value	Standard error	*T*-value	*P*-value
Intercept	6.67	0.89	7.48	< 0.001
Mean temperature	−0.153	0.0392	−3.902	** < 0.001**
Mean wind speed	−0.076	0.0343	−2.219	**0.03**
Trap type (Vavoua)	2.084	0.634	3.284	**0.002**
Distance of traps to dung	−0.007	0.0035	−2.082	**0.04**

A quasi-Poisson distribution was used. Bold values indicate statistical significance

Conversely, in the southern sites of Gran Canaria (Table 3; Model 2), temperature was the only variable showing a significant effect, again with a negative relationship with abundance (*P* = 0.01), while the remaining variables showed no significant effects.

**Table 3:** Model 2: GLMM results for locations in southern Gran Canaria, assessing the effect of mean temperature, mean wind speed, trap type (Nzi as reference vs. Vavoua), and distance between traps and dung accumulations on the number of *Stomoxys calcitrans* specimens collected per trap

	Value	Standard error	*T*-value	*P*-value
Intercept	7.27	1.462	4.974	< 0.001
Mean temperature	−0.156	0.058	−2.66	**0.01**
Mean wind speed	0.043	0.028	1.51	0.138
Trap type (Vavoua)	0.344	0.256	1.346	0.186
Distance of traps to dung	−0.007	0.007	−0.966	0.3402

A quasi-Poisson distribution was used. Bold values indicate statistical significance

In Lanzarote (Table 4; Model 3), the GLMM did not detect any significant effects of the climatic variables on abundance. However, distance to dung accumulations showed a significant positive relationship with abundance, suggesting a pattern opposite to that observed in northern Gran Canaria (*P* = 0.03).

**Table 4:** Model 3: GLMM results for locations in Lanzarote, assessing the effect of mean temperature, mean wind speed, trap type (Nzi as reference vs. Vavoua), and distance between traps and dung accumulations on the number of *Stomoxys calcitrans* specimens collected per trap

	Value	Standard error	*T*-value	*P*-value
Intercept	4.235	1.88	2.25	0.029
Mean temperature	−0.094	0.074	−1.26	0.22
Mean wind speed	0.046	0.048	0.97	0.342
Trap type (Vavoua)	−0.392	0.38	−1.03	0.308
Distance of traps to dung	0.016	0.007	2.17	**0.03**

A quasi-Poisson distribution was used. Bold values indicate statistical significance

Finally, in Fuerteventura (Table 5; Model 4), temperature again showed a significant negative effect on abundance, consistent with the results obtained in Gran Canaria (*P* = 0.01). In addition, trap type had a significant effect, with the Nzi trap showing higher capture efficiency on this island (*P* = 0.02).

**Table 5:** Model 4: GLMM results for locations in Fuerteventura, assessing the effect of mean temperature, mean wind speed, trap type (Nzi as reference vs. Vavoua), and distance between traps and dung accumulations on the number of *Stomoxys calcitrans* specimens collected per trap

	Value	Standard error	*T*-value	*P*-value
Intercept	15.7	4.05	3.89	0.0013
Mean temperature	−0.38	0.13	−2.88	**0.01**
Mean wind speed	0.028	0.06	0.45	0.657
Trap type (Vavoua)	−1.254	0.505	−2.48	**0.02**
Distance of traps to dung	−0.042	0.04	−0.99	0.33

A quasi-Poisson distribution was used. Bold values indicate statistical significance

Model explanatory power varied across analyses. Models 1 and 4 showed relatively high marginal *R*^2^ values (0.63 and 0.53, respectively), which were identical to their conditional *R*^2^ values, indicating that most of the explained variance was attributable to fixed effects. In contrast, Model 2 showed a substantial increase from marginal *R*^2^ (0.33) to conditional *R*^2^ (0.51), highlighting the importance of random effects. Model 3 showed comparatively low explanatory power (marginal *R*^2^ = 0.19; conditional *R*^2^ = 0.26), indicating limited variance explained by both fixed and random components.

## Discussion

The aim of this study was to determine the presence and abundance of potential vectors of *T. evansi* and to analyse the climatic and site-related factors affecting their distribution. Together with the recently published atlases on surra and its potential vectors in Spain [30, 46], this work will contribute to improving the understanding of the epidemiology of this disease, especially in the Canary Islands. The introduction of the disease into the Canary Islands was probably because of the importation of carrier animals from Morocco and Mauritania, since the dromedary population in the Canary Islands is derived primarily from animals imported from these countries [47]. From the description of the first case [21] to the point the disease was considered under control [29], almost 25 years elapsed. Knowledge of the ecology of disease vectors is essential to the design of prevention and control plans to avoid future outbreaks.

Among the three haematophagous dipteran species identified, *S. calcitrans* was the most abundant and widely distributed across all sampling sites. This species has a global distribution and is known to be a mechanical vector of several pathogens, including *Bacillus anthracis* and *Coxiella burnetii* (Q fever), as well as a cyclical vector of *Habronema* spp., among others [48]. In the Canary Islands, its presence has been confirmed on all islands [34]. The role of *S. calcitrans* in transmitting *T. evansi* has been proposed by several authors [3, 48], and its presence was confirmed in a high-prevalence surra zone on Gran Canaria [28]. An entomological survey conducted in an area geographically close to the present study’s A1L1 site provided valuable monthly data on the dynamics of *S. calcitrans* over a 1-year period [28]. For this survey, an Nzi trap was deployed on a dromedary farm, capturing only 993 specimens. The annual peak in abundance occurred in November, while the lowest captures were recorded in February.

One of limitation of the current study is its temporal scope. Although the study covered 3 islands and 10 locations, it does not provide a continuous picture of annual variation, because sampling was restricted to 4 specific months over 2 years. Comparisons between our data and previous studies [28] do not consistently reveal similar trends, possibly because of behavioural differences in insect populations between sites, despite their geographical proximity, climatic differences, and changes in land use, or host presence, among others. However, this remains speculative because of the differences in the sampling methodologies used.

As noted in the previous section, GLMM analyses revealed significant associations between climatic and spatial parameters and the abundance of *S. calcitrans*. Understanding the species’ ecological requirements (particularly its thermal preferences) along with its developmental thresholds and the climatic characteristics of the Canary Islands provides insights into how climatic variables affect the species abundance in the region. Optimal development and survival occur between 20 and 25 ºC, whereas temperatures < 10.2 ºC or > 33.5 ºC are considered limiting [17, 49]. In Gran Canaria, particularly at the northern sampling sites, the highest temperature recorded over the 2-year sampling period was 40 ºC (summer of 2023), while the lowest reached 6 ºC in winter months. Overall, the general trend indicated mean temperatures ranging from 18 to 23 ºC. In contrast, southern locations exhibited higher values, with a maximum of 45 ºC (summer of 2023) and a minimum of approximately 14 ºC, with mean temperatures generally ranging between 24 and 26 ºC. In Lanzarote, the highest recorded temperature was 37 ºC (summer 2023), while the lowest was 9 ºC in winter, with an overall mean trend between 18 and 23 ºC. Similarly, in Fuerteventura, the maximum temperatures reached 41 ºC (summer 2023), with a minimum of 10 ºC, and a general mean range between 21 and 24 ºC. Overall, these data suggest that mean temperature ranges across all the islands are generally suitable for *S. calcitrans*. However, during certain periods of the year (particularly summer and winter), temperatures may exceed the upper or lower thresholds for this species. Notably, such extremes can vary between years and locations, and they may include exceptional events, such as the unusually high temperatures recorded in the summer of 2023, which were not observed again during the remaining months.

Conversely, the presence of strong winds was negatively associated with abundance. Although this effect was statistically significant only in northern Gran Canaria, it is ecologically plausible, as strong winds can substantially hinder these dipterans from reaching a host or a trap. The highest mean wind speed recorded during the sampling period reached 30 km/h, specifically in southern Gran Canaria. In the northern areas, the maximum mean value was 27 km/h, with an overall trend ranging between 8 and 13 km/h throughout the study period. A similar pattern was observed in the south, although with slightly higher sustained values, generally between 8 and 15 km/h. In Lanzarote, the highest mean wind speed was 24 km/h, while most recorded speedss consistently ranged from 12 to 20 km/h. Likewise, in Fuerteventura, the maximum mean value reached 26 km/h, with most observations falling within the 12–20 km/h range. Overall, these patterns suggest that the statistical significance observed in the north of Gran Canaria may be present in the other locations examined, as similar or even higher values were obtained in some of them. Therefore, further research is required to better understand this relationship, particularly by integrating additional spatial variables in future analyses. Despite these meteorological trends, the presence of *S. calcitrans* appears to be year-round, with no clear seasonal absence, likely favoured by the islands’ stable and thermally suitable conditions for much of the year.

In addition to the two meteorological parameters included in the GLMMs, the relatively high values of cumulative mean precipitation (not incorporated into the models because of their correlation with temperature) appear to be associated in some locations’ graphs (mainly A1L2, A1L3, A1L4, A2L2, and A2L3) with subsequent peaks of *S. calcitrans* 1 to 4 months after rainfall events. Confirming this pattern will require more extensive year-round sampling. With only four data points per year available, the relationship, although visually evident, cannot be tested with sufficient robustness.

Concerning trap proximity to dung heaps, a clear disparity was observed: in northern Gran Canaria, abundance increased as traps were placed closer to dung heaps, whereas in Lanzarote, the opposite trend was detected. The former pattern is ecologically consistent, given that *S. calcitrans* primarily develops in organic matter such as manure. However, the latter does not contradict this, as alternative substrates or site-specific factors may favour development away from dung heaps, especially if the substrate becomes too dry and therefore not suitable for larval development. One possible explanation, based on observations at some sampled sites, is that livestock are allowed to roam freely across different areas of farms (outside their enclosures) where they graze on nearby vegetation and defecate (particularly small ruminants). Another example involves camel farms on the islands, where animals are used for tourism (especially for tours) and are trained outside the farms, are walked through designated areas, and defecate along the way, thus dispersing potential substrates similarly to small ruminants. Although the study areas did not appear to contain other substrates (e.g., agricultural waste) that could support larval development in the surrounding environment, the potential relationship between other organic matter and the species’ abundance should be examined under local conditions in future research.

Regarding trap performance across locations, it is noteworthy that the sampling design generally included more Vavoua than Nzi traps (2:1), a disproportion that may bias statistical outcomes and fail to accurately reflect their true relative effectiveness. Moreover, the specific placement of traps could influence captures: Nzi traps were positioned in more open areas due to their morphology and primary use for tabanid collection, whereas Vavoua traps target stomoxyines [50]. This supports the observed pattern in northern Gran Canaria, where Vavoua traps captured more *S. calcitrans*. Conversely, the unexpected outcome in Fuerteventura may therefore be attributable to site-specific placement decisions. In this regard, the specificity of the traps for capturing *S. calcitrans* was low and similar between both trap types (approximately 40%), considering the high number of non-haematophagous muscids collected. Consequently, considering all these factors, accurately assessing the true performance of each trap across the sampled locations would require a standardised methodological approach.

Concerning the variation in the number of *S. calcitrans* among the different islands, beyond the evident fact that some islands had more sampling sites than others, we suggest that the presence of crops (particularly angiosperm flora) may favour the abundance of these vectors. For example, at location A1L4, one specimen was observed feeding on nectar. Such behaviour could enhance the survival of certain populations during unfavourable periods when access to hosts may be limited. For example, during periods when climatic conditions are not favourable for adult activity, such as those characterized by high wind speeds, rainfall, or temperatures that may reduce insect mobility, nectar feeding could represent a relevant source of energy for short-term survival. This assumption could be supported by the fact that *S. calcitrans* commonly seeks refuge in both livestock facilities and vegetation [18]; therefore, under unfavourable conditions that limit access to hosts, such situations may arise. In this context, some studies have shown that nectar supplementation in blood-feeding individuals may even enhance larval emergence [51]. In the specific case of the Canary Islands, however, this hypothesis requires further investigation. Capturing postprandial individuals during the present sampling efforts may help shed light on their feeding habits and the specific periods during which this species might rely on nectar as an additional food source. The presence of different host species may also affect abundance, as observational evidence indicates that cattle, equids, and dromedaries could favour higher numbers of *S. calcitrans* in the area. Nevertheless, these hypotheses require confirmation and further investigation. In this regard, applying metabarcoding techniques to preserved specimens could provide valuable insights into the feeding habits and host preferences of this species across different sampling sites [18, 48].

From an applied perspective, the results of this study may support the design of targeted surveillance and control strategies for *S. calcitrans* in the Canary Islands. The influence of temperature and wind suggests that monitoring efforts should be adapted to local climatic conditions. In addition, the association with dung accumulations highlights the importance of manure management as a potential control measure. Therefore, surveillance programmes should prioritise high-density livestock areas and consider environmental conditions that may favour vector abundance.

As previously noted, the presence of the other two haematophagous dipteran species was occasional. *Tabanus cordiger* was first reported in the Canary Islands in 1936 by entomologist Richard Frey (initially designated as *Tabanus fortunatus*) [35]. According to the Canary Islands Biodiversity Data Bank (https://www.biodiversidadcanarias.es/biota/), its current known distribution is restricted to Tenerife and Fuerteventura. In the present study, a single female specimen of *T. cordiger* was recorded at site A3L2 on Fuerteventura.

Given the limited and outdated information on Tabanidae in the archipelago [35], further entomological research is needed to assess the current abundance, diversity, and distribution. This is particularly relevant considering their potential mechanical or biological vector roles for pathogens of veterinary and medical importance, such as *Anaplasma* spp., equine infectious anaemia virus, and filariform nematodes [16]. Clarifying their vectorial capacity and epidemiological relevance under insular conditions should be prioritised in future surveillance programmes.

In contrast, the hippoboscid fly *P. canariensis* is considered an obligate ectoparasite of birds, particularly domestic pigeons, and a known vector of the protozoan *Haemoproteus columbae* Kruse, 1890 [19, 52]. In this study, the species was recorded at eight sites: four in Gran Canaria, two in Lanzarote, and two in Fuerteventura. The highest number of individuals (*n* = 49) was captured at site A3L2. The presence of *P. canariensis* is likely linked to the high pigeon density observed near the locations. Further investigation is needed to determine the extent to which this species is attracted to the traps used in the study as well as to evaluate its potential opportunistic behaviour. Although *P. canariensis* is typically bird-specific, its proximity to livestock in shared environments raises questions about its ability to feed facultatively on mammals. Accordingly, several postprandial specimens were preserved in ethanol for future biomolecular analyses. These studies will aim to identify host-vector interactions and assess the possible presence of other pathogens, thereby enhancing our understanding of the species’ broader epidemiological relevance.

## Conclusions

This study represents a substantial contribution to the entomological surveillance of potential mechanical vectors of *T. evansi* in the Canary Islands. Although no current circulation of *T. evansi* has been detected on the investigated islands, the persistent presence of haematophagous dipterans, particularly *S. calcitrans*, which appears to be the predominant species of potential epidemiological relevance, underscores the importance of continued vector monitoring and ecological characterisation.

In this regard, further research and long-term continuous field studies are needed (ideally spanning full calendar years, with weekly sampling and precise weekly climate records) to provide a comprehensive, year-round understanding of *S. calcitrans* population dynamics. In addition, expanded sampling across the remaining islands of the archipelago (those within the province of Santa Cruz de Tenerife) is necessary to assess the species’ abundance and behavioural patterns more accurately.

## Data Availability

Data supporting the main conclusions of this study are included in the manuscript.
